# Intraoperative radiotherapy as an immediate adjuvant treatment of rectal cancer due to limited access to external-beam radiotherapy

**DOI:** 10.1186/s13014-020-1458-y

**Published:** 2020-01-10

**Authors:** Sergey Potemin, Jens Kübler, Ivan Uvarov, Frederik Wenz, Frank Giordano

**Affiliations:** 1 Department of Colorectal Surgery, Regional Oncological Center of Krasnodar, Krasnodar, Russia; 2 Department of Radiation Oncology, Universitätsmedizin Mannheim, Medical Faculty Mannheim, Heidelberg University, Theodor-Kutzer-Ufer 1-3, 68167 Mannheim, Germany

**Keywords:** Intraoperative radiotherapy, External beam radiotherapy, Rectal cancer

## Abstract

**Background:**

Neoadjuvant external-beam radiotherapy (EBRT) with concomitant chemotherapy is the current standard-of-care for locally-advanced rectal cancer. Intraoperative radiotherapy (IORT) is to date only recommended for pelvic recurrences or incompletely resectable tumors. We here report on patients with stage II/III rectal cancer that were treated with IORT in a regional Russian university center due to limited access to EBRT.

**Methods:**

We retrospectively analyzed data from patients that were diagnosed with locally-advanced rectal cancer and underwent surgery from December 2012 to October 2016 at a regional oncological center in Russia (Krasnodar). During this period, access to EBRT was limited due to a temporary lack of a sufficient number of EBRT facilities. Patients unable to travel to a distant radiotherapy site received IORT alone, those that could travel received neoadjuvant external beam (chemo-) radiotherapy. Factors of interest were tumor stage, tumor differentiation, resection status, surgery type and neoadjuvant or adjuvant chemotherapy. We assessed local progression-free survival (L-PFS), PFS and overall survival (OS).

**Results:**

A total of 172 patients were included in this analysis. Of those, 92 (53.5%) were treated with IORT alone (median dose: 15 Gy [8.4–17 Gy]) and 80 (46.5%) received both neoadjuvant EBRT (median dose: 50.4 Gy [40–50.4 Gy]) and IORT (median dose: 15 Gy [15–17 Gy]). The median age was 65 years [33–82]. The median follow-up was 23 months [0–63 months]. The incidence of toxicity was low in both groups with an overall complication rate of 5.4%. Local PFS at 4 years was comparable with 59.4% in the IORT group and 65.4% in the IORT/EBRT group (*p* = 0.70). Similarly, there was no difference in OS or PFS (*p* = 0.66, *p* = 0.51, respectively).

**Conclusions:**

IORT is a valuable option for patients with locally-advanced rectal cancer in the absence of access to EBRT.

## Background

Despite the advances made in recent years, treatment of advanced rectal cancer remains challenging. Complete surgical excision of the tumor and margins provide the cornerstones of treatment. However, patients require a multimodal approach involving surgery, chemotherapy and radiation therapy [1–4]. In locally-advanced rectal cancer, neoadjuvant chemoradiotherapy has become standard-of-care. Compared to preoperative chemotherapy alone, preoperative chemoradiotherapy can further reduce the rate of local recurrence, decrease tumor volume and improve the resection rate [5]. Whereas completion of oncologic treatment over several weeks is standard of care in most industrialized nations, patients in emerging countries frequently struggle to obtain long-term continuous therapy due to a lack of sufficient numbers of treatment facilities. It is therefore worth exploring less resource intense alternatives to standard of care long-course neo-adjuvant chemoradiotherapy.

EBRT is known to significantly improve outcome in locally advanced rectal cancer, however based on the necessity of fractionated application, it requires adequate availability, infrastructure and compliance of the patients. One approach to provide radiation therapy to the tumor bed in the absence of access to EBRT is intraoperative radiotherapy (IORT). In IORT, a single dose of radiation (usually 8–20 Gy) is delivered to the region that is considered to be at risk for recurrence. It allows direct visualization of the tumor bed, maximizes the biological effect of a high dose of irradiation and minimizes the risk of complications in the surrounding healthy tissues, since organs at risk can be shifted out of the radiation field or shielded during the procedure [6, 7]. Furthermore, IORT can be provided at low costs that are usually only a fraction of the acquisition and maintenance of a classical linear accelerator used for EBRT.

A delayed technical repair in a regional Russian university center caused a temporary lack of an EBRT facility. Consequently, the amount of radiotherapy sessions that could be provided decreased and several patients were forced to travel to a distant treatment center. Patients in need of radiotherapy but unable to travel due to limited traffic connections were then treated with sole IORT.

Over the past decades, a large number of groups have reported improved outcomes of IORT added to neoadjuvant EBRT or chemoradiotherapy [8–11]. To date no data are available describing IORT as exclusive adjuvant treatment in locally-advanced rectal cancer, since EBRT is an approved and very effective method that is usually broadly available. However, alternative therapeutic strategies might be considered in centers that do not have sufficient resources to administer optimum treatment.

We here report on a total of 172 patients with locally-advanced rectal cancer (stage II or III). 80 patients underwent surgery and IORT after neoadjuvant EBRT (with or without chemotherapy) and 92 patients received surgery and IORT without neoadjuvant treatment.

## Patients and methods

### Patient collective

Patients diagnosed with histologically confirmed locally advanced (stage II-III, pT3–4 or pN+) rectal cancer that underwent radical surgery with IORT from December 2012 to October 2016 at a regional oncological center in Krasnodar, Russia, were retrospectively analyzed. During this period, there was only limited access to radiotherapy facilities until a new local facility became available in 2017. We included adult patients independently of resection status (R0, R1, R2), primary cancer site (upper, middle or lower rectum) or tumor differentiation. Patients with previous history of irradiation, second tumors or concurrent distant metastasis were excluded.

### Surgery and IORT

Surgery was carried out according to standard protocols, performing either anterior rectal resection (ARR) with total mesorectal excision (TME), abdominoperineal resection or Hartmann’s procedure. After specimen excision, careful macroscopic assessment was performed to determine and mark the area with the highest risk of involvement. A circular border surrounding the resected tumor margins was marked by stitching to define the target volume. For IORT, the Intrabeam® system (Carl Zeiss Meditec AG, Oberkochen, Germany) was employed using spherical applicators with diameters of either 4.5 cm or 5 cm. A single dose of 10–20 Gy was delivered, whereas the dose was dependent on the proximity of surrounding risk structures (e.g. the sciatic plexus). In all cases, IORT was performed immediately following tumor resection. Prior to IORT, the small intestine was covered with gauze and moved in the cranial direction using an extractor. In certain cases, tourniquets were applied to the ureters and these were separated laterally from the radiation field. The vascular fascicles and ureters were protected with special sterile plates and dry gauze stacked on the pelvic sidewalls. In patients who underwent ARR, the rectal stump was protected in the same way. After IORT, the surgical intervention continued in the habitual way. For sphincter-sparing operations, an anastomosis was created. In case abdominoperineal resection was performed, the perineal wound was closed. In all cases with an anastomosis, protective ileostomy was performed.

### Neoadjuvant and adjuvant treatment

Only patients willing and able to travel to a distant radiotherapy site in spite of limited public transportation received neoadjuvant EBRT with or without concomitant fluoropyrimidine-based chemotherapy, i.e. continuous infusion 5-FU with leucovorin plus oxaliplatin (FOLFOX regimen) or irinotecan (FOLFIRI regimen) if positive lymph nodes or incomplete resection were suspected. All medically fit patients received adjuvant chemotherapy with 3–6 cycles of FOLFOX or FOLFIRI after surgery. EBRT was performed using a linear accelerator and 2D-conformal radiotherapy delivering up to 50.4 Gy in daily fractions of 1.8 Gy. In case neoadjuvant treatment was applied, surgery was performed 6–8 weeks after completion of (chemo-) radiotherapy.

### Follow-up

Follow-up was performed every 3 to 6 months and consisted of routine physical examination, abdominal ultrasound, abdominal/pelvic CT imaging, MRI and biochemical parameters (CEA, CA19–9) for up to 5 years. Local recurrence was defined as any histologically proven evidence of rectal cancer recurrence in the small pelvis including recurrence at the site of anastomosis and perineal wound. Distant recurrence was defined by any histological, morphological and clinical evidence of distant metastases including lymph nodes.

### Statistical analyses

Patient characteristics and tumor-related variables were compared by using the Wilcoxon rank sum test, the Kruskal-Wallis test or the t-test for independent samples. The χ^2^ test for proportions was performed for comparison of categorical data within each cohort and between-group differences. Four-year survival rates and progression-free survival rates were determined by the Kaplan-Meier method. Freedom from progression was defined as time span from surgery until progression at any site, whereas death by any cause was not considered an event for time to progression (TTP). Progression-free survival (PFS) was defined as the length of time from surgery until progression or death by any cause. Local progression was defined as any histologically proven evidence of rectal cancer recurrence in the small pelvis including recurrence at the site of anastomosis and perineal wound. Overall survival was defined as time span from surgery until death by any cause. Univariate comparisons were performed with the log-rank or Breslow test. Any difference of variables with associated *p*-values lower than 0.05 was considered statistically significant.

## Results

### Patient characteristics

172 patients with stage II or III rectal cancer who underwent surgical resection and IORT or resection and neoadjuvant EBRT and IORT between December 2012 and October 2016 were included in this analysis (Table 1). The median age was 65 years (range: 33–85 years), the sex ratio was well balanced (53.3% male patients in the IORT group and 48.8% in the IORT/EBRT group). In the IORT group 83.7% of the patients were initially diagnosed stage II rectal cancer and 16.3% stage III. In the IORT/EBRT group it was 51.3 and 48.8%, respectively (*p* <  0.0001). In the IORT group primary cancer site was found to be the middle third rectum in most patients (57.6%), whereas the majority of patients (70.0%) in the IORT/EBRT group were diagnosed with lower third rectal cancer (*p* <  0.0001). In 169 patients (98.2%) adenocarcinoma was histologically confirmed, in 3 patients the specific tumor entity could not be determined. Most patients in both groups were diagnosed with grade 2 (moderately differentiated) tumors (85.9% in the IORT group and 90% in the IORT/EBRT group).

**Table 1:** Demographic and disease characteristics of the patients (*N* = 172)

Characteristic	IORT Group	IORT+EBRT Group	*P*
no. (%)	*N* = 92 (53.5)	*N* = 80 (46.5)	
Follow-up duration - median (range) - months	25 (0.9–62.5)	22 (0–57.7)	0.52
Age			0.67
Median (range) - yr	65.5 (38.0–83.0)	65 (33.0–85.0)	
< 65 yr - no (%)	40 (43.5)	38 (47.5)	
Male sex - no. (%)	49 (53.3)	39 (48.8)	0.55
Primary Cancer Site - no. (%)			< 0.0001
Upper Third Rectum (incl. rectosigmoid)	17 (18.5)	5 (6.3)	
Middle Third Rectum	53 (57.6)	19 (23.8)	
Lower Third Rectum	22 (23.9)	56 (70.0)	
neoadjuvant EBRT Dose [Gy] - no. (%)			
50,4	0	79 (98.7)	
< 50,4	0	1 (1.3)	
Pathologic Tumor Stage - no. (%)			
T			0.047
T2	2 (2.2)	2 (2.5)	
T3	82 (89.1)	59 (73.8)	
T4a	7 (7.6)	14 (17.5)	
T4b	1 (1.1)	5 (6.3)	
N			0.0001
N0	78 (84.8)	41 (51.3)	
N1	11 (12.0)	25 (31.3)	
N2	3 (3.3)	14 (17.5)	
Stage Group - no. (%)			0.0001
II	77 (83.7)	41 (51.3)	
IIA	72 (87.3)	32 (40.0)	
IIB	5 (5.4)	8 (10.0)	
IIC	0	1 (1.3)	
III	15 (16.3)	39 (48.8)	
IIIA	8 (8.7)	13 (16.25)	
IIIB	7 (7.6)	24 (30.6)	
IIIC	0	2 (2.5)	
Histology - no. (%)			0.5
Adenocarcinoma	91 (98.9)	78 (97.5)	
Squamous cell carcinoma	1 (1.1)	1 (1.2)	
Mucinous carcinoma with ring cell components	0	1 (1.2)	
Differentiation - no. (%)			0.25
Well differentiated (G1)	4 (4.3)	0	
Modarately differentiated (G2)	79 (85.9)	72 (90.0)	
Poorly differentiated (G3)	8 (8.7)	6 (7.5)	
Grade cannot be assessed (Gx)	1 (1.1)	6 (7.5)	
Tumor size			0.02
median (range) - cm	4.0 (1.5–9)	5.0 (2.0–8.0)	
< 2.5 cm - no. (%)	5 (5.4)	2 (2.6)	
2.5–4.0 cm - no. (%)	43 (46.7)	29 (36.3)	
> 4.0 cm - no. (%)	44 (47.8)	49 (61.3)	
Resection - no. (%)			0.0001
High anterior resection	15 (16.3)	4 (5.0)	
Low anterior resection	63 (68.5)	15 (18.8)	
Ultra Low anterior resection	3 (3.3)	1 (1.3)	
Abdomnial perineal excision	8 (7.9)	63 (75.0)	
Hartmann’s Procedure	3 (3.3)	1 (1.3)	
Resection status - no. (%)			0.2
R0	89 (96.7)	73 (91.3)	
R1	3 (3.3)	5 (6.3)	
R2	0	2 (2.5)	
Stoma - no. (%)			0.0001
End Colostomy	20 (21.7)	60 (75.0)	
Loop Colostomy	66 (71.7)	19 (23.8)	
Loop Ileostomy	5 (5.4)	1 (1.3)	
none	1 (1.1)	0	
IORT - median (range)			
Dose on applicator surface (Gy)	15 (15.0–17.0)	15 (8.4–17)	0.1
Treatment Time (min)	34 (25.0–38.0)	34 (15.0–38.0)	0.64
Applicator Size (cm)	5 (4.5–5.0)	5 (4.5–5.0)	0.68
Days to discharge after surgery - median (range)	16 (5.0–25.0)	16 (9.0–42.0)	0.4
Chemotherapy - no. (%)			0.0001
any	10 (10.9)	37 (46.3)	
both	0	17 (21.3)	
neoadjuvant	0	20 (25.0)	
adjuvant	10 (10.9)	34 (42.5)	

### Treatment

92 patients (53.5%) were treated with IORT alone with a median dose of 15 Gy prescribed to the applicator surface. The median IORT treatment time was 34.0 min. Eighty patients (46.5%) received both neoadjuvant EBRT (median dose 50.4 Gy) and IORT. Only 2 (2.6%) patients received less than 50.4 Gy.

Only a reduced percentage of patients who received radiotherapy with preoperative intention also received neoadjuvant chemotherapy (20/81, 24.7%). Furthermore, a very limited percentage in both subgroups but above all in the IORT subgroup received adjuvant chemotherapy (10.9% vs. 42.5%).

The majority of patients (68.5%) in the IORT group were treated with low anterior resection while 63 patients (75%) received abdominoperineal resection in the IORT/EBRT group (*p* < 0.0001). Margins were positive in 10 patients (IORT group: 3.3% R1 resection; IORT/EBRT group: 6.3% R1 resection, 2.5% R2 resection). More patients in the IORT group received loop colostomy (71.7%) than end colostomy, whereas the majority in the IORT/EBRT group was treated with end colostomy (75.0%).

### Adverse events

The overall complication rate for all 172 patients was 5.4%. Postoperative infections were observed in 9 patients as follows: abdominal wound infection in 3 cases (1.7%), perineal wound infection in 6 cases (3.5%). Bladder atony was seen in one patient. One case of colorectal anastomosis leakage from 109 patients was recorded. There was no postoperative gastrointestinal or genitourinary toxicity. Furthermore there were no IORT specific complications. The median time to discharge after surgery was 16 days (range: 5.0–42 days).

### Recurrence and survival

**Fig. 1:**
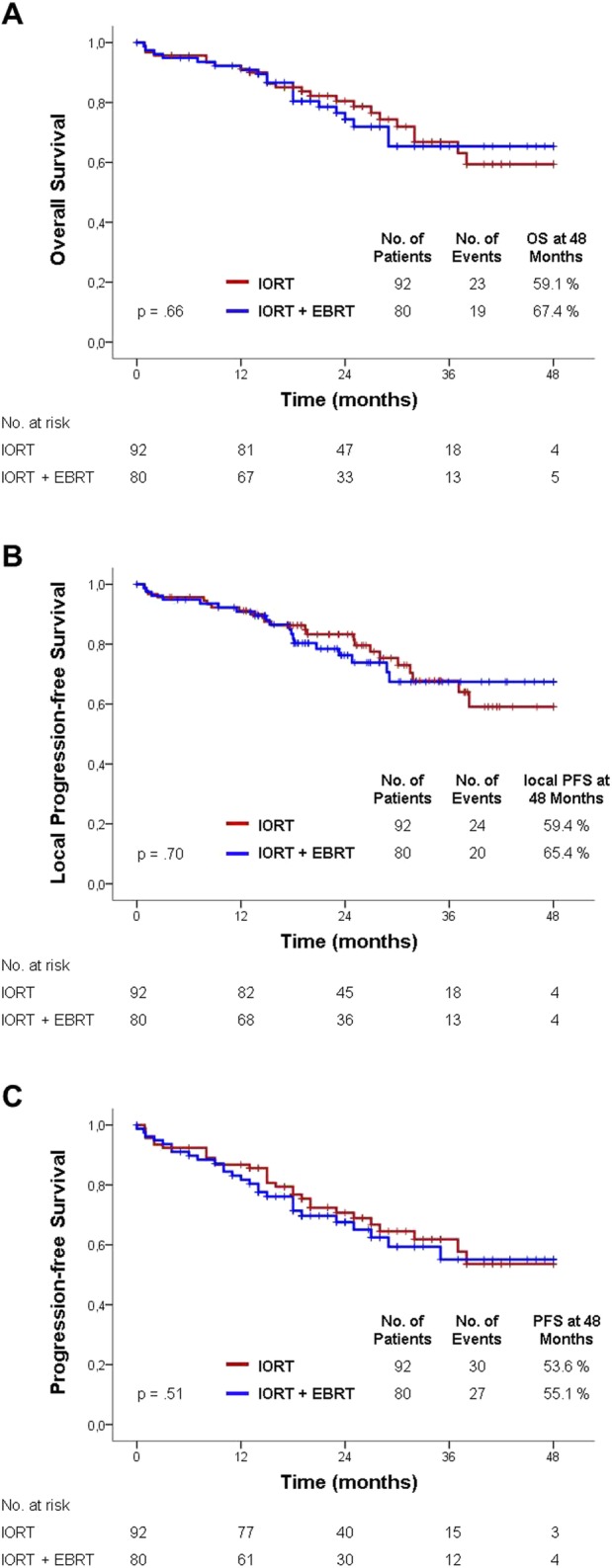
Outcomes after IORT or IORT+EBRT. **a** Overall survival. **b** Local progression-free survival. **c** Progression-free survival

The median follow-up period was 25 months for the IORT group and 22 months for the IORT/EBRT group (*p* = 0.52). Tumor recurrences were observed in a total of 25 patients. Three patients had a local recurrence, 23 showed distant recurrences. There was no significant difference between the IORT and IORT/EBRT group in time to local (*p* = 0.68) or any progression (*p* = 0.26). There was no significant difference in overall survival (OS) between both groups (59.1% vs. 67.4%, *p* = 0.66). Progression-free survival at 4 years was 53.6 months in the IORT group and 55.1 months in the IORT/EBRT group (*p* = 0.51). Local progression-free survival at 4 years was 59.4% in the IORT group and 65.4%in the IORT/EBRT group (*p* = 0.70) (Fig. 1).

We furthermore analyzed subgroups of patients with stage II or III rectal cancer separately and saw no statistically significant difference in overall survival between the IORT and IORT/EBRT groups (*p* = 0.63 and *p* = 0.98, respectively; Additional file 1: A1). Also, freedom from local or any progression was not statistically different between both treatment groups in stage II (*p* = 0.5 and *p* = 0.19, respectively) and stage III (*p* = 0.23 and *p* = 0.75, respectively).

## Discussion

Neoadjuvant chemoradiotherapy over several weeks is the standard of care therapy for locally advanced rectal cancer [12, 13]. According to the latest recommendations of the NCCN 2019 guidelines IORT should be considered for very close or positive margins after resection, as an additional boost, especially for patients with T4 or recurrent cancers [14, 15]. It improves local control and reduces the incidence of toxicity but does not impact on overall survival.

In recent years, IORT has become increasingly popular in oncologic surgery and has been introduced as boost or sole treatment into many multimodal treatment protocols [16]. Adjuvant EBRT is usually not applied before at least 2 weeks after surgery due to wound healing processes. During this period, residual tumor cells may be able to re-grow and/or migrate and on, the long run, cause local relapses. In this regard, IORT offers the advantage of instantaneous “sterilization” of the tumor bed, which was proven to be highly efficient in a wide variety of intra-abdominal tumours, recurrent colorectal cancers, recurrent gynaecological cancers, and soft-tissue tumours breast cancer, brain tumors and soft-tissue sarcoma [17–20]. A number of mono-institutional trials demonstrated improved local control of rectal cancer after IORT even in the era of neoadjuvant therapy [10], whereas specifically patients with microscopically-involved circumferential resection margins (CRM) may benefit most [9, 21–23]. In a systematic review of 29 studies including more than 3000 patients with locally advanced primary or recurrent colorectal cancer, IORT was even seen to improve local control, disease-free survival and overall survival without increasing urological and anastomotic complication rates [24]. Likewise, Alberda et al. found that patients with a microscopically-involved CRM treated with IORT had a significantly better cumulative 5-year local recurrence-free survival compared to patients treated without IORT (84 vs. 41%, *p* = 0.01) [9]. Moreover, in multivariate analyses, IORT was an independent factor that reduces the local recurrence rate. These data suggest that IORT may be indicated in tumors with close or positive microscopic margins. The limitations of the study reveal different scenarios that could be modified and evaluated in the future, and thus be able to draw conclusions that could modify our clinical practice in the event that we do not have all the necessary resources to be able to offer a therapeutic strategy of the best possible quality.

In most previous studies, IORT was delivered using electrons [21, 22, 25–27] or, less frequently, with high-dose rate brachytherapy (HDR BT) [28, 29]. Irradiation with electron beams required re-location of the intubated patient from the operation room into the radiation treatment bunker and placement of a cone into the situs to deliver forward-scattering electrons, which may explain why some trials did not detect a benefit [25]. We followed the Cleveland Clinic method [7] and used a mobile x-ray device that delivers radiotherapy differently: first, IORT was delivered through spherical applicators, which provide isotropic dose distributions that achieve a high (er) degree of target volume coverage than forward-scattering beams. Second, we used low-energy (max 50 kV) x-rays, which, due to exponential attenuation in matter, penetrate only few millimeters into the tissue, thereby allowing to achieve high doses to the tumor bed with very little doses to organs at risk. Third, low-energy x-rays have a higher relative biological effectiveness due to their increased linear energy transfer (i.e. more DNA double strand breaks occur per distance of penetrated tissue) than conventional high-energy photons or electron beams [30].

This study is retrospective in nature and thus it has several limitations: first, 5 year OS rates and recurrence rates in locally-advanced rectal cancer were reported to be 70–80% and 10–20%, respectively [13, 31, 32]. Survival rates at 4 years in this trial were ranging around 60% and were thus lower. However, local recurrence rates were around 3%, which may indicate that there were more non-cancer related deaths due to a generally lower life expectancy in Russian population (64.7 for males and 76.3 years for females in Russia versus 78.7 for males and 83.4 for females years in Germany) [33, 34]. Second, imbalances in the collective with stage III patients in the IORT only group appeared to limit the interpretation of the impact of IORT alone. We tackled this by performing subgroup analyses of stage II and III, where we also detected no statistically significant difference in OS, freedom from local progression or any progression, indicating that IORT has an impact as sole treatment. Imbalance concerning tumor region (IORT-group: middle-third rectum 58%, IORT/EBRT-group lower-third rectum 70%) and subsequently in the surgical procedure (IORT-group: LAR 68%, IORT/EBRT-group: APR 75%) might additionally influence outcome. Yet, independently from a comparison between different stages and treatments our results demonstrate that there might be a sufficient local control in stage II patients who were treated with IORT and without neoadjuvant EBRT. Third, although we did not observe severe complications after IORT, the median follow-up of 5 years might be too short to give a valid statement specifically on late toxicity (such as neuropathy). Nevertheless, the toxicity profile is very low as seen in previous studies such as the French randomized trial [25] or the Cleveland Clinic experience [7]. No relevant differences were observed either in terms of surgical complications or in hospital stay.

## Conclusions

Intraoperative radiotherapy represents a valuable treatment option in stage II/III rectal cancer patients in the absence of EBRT.

## Supplementary information

**Additional file 1 A1.** Stage II or III rectal cancer subgroups. (A, B) Overall survival. (C, D) Freedom from local progression. (E, F) Freedom from local or distant progression.

## Data Availability

The datasets used and/or analyzed during the current study are available from the corresponding author on reasonable request.

## Abbreviations

ARR: Anterior rectal resection
CRM: Circumferential resection margins
EBRT: External beam radiotherapy
FOLFIRI: 5-FU with leucovorin plus irinotecan
FOLFOX: 5-FU with leucovorin plus oxaliplatin
HDR BT: High-dose rate brachytherapy
IORT: Intraoperative radiotherapy
L-PFS: Local progression-free survival
OS: Overall survival
PFS: Progression-free survival
TME: Total mesorectal excision
TTP: Time to progression
