# Supplementary figures and images for: Intraoperative radiotherapy as an immediate adjuvant treatment of rectal cancer due to limited access to external-beam radiotherapy
Source: Radiat Oncol. 2020 Jan 10;15:11. doi: 10.1186/s13014-020-1458-y (PMC6954580; doi:10.1186/s13014-020-1458-y)

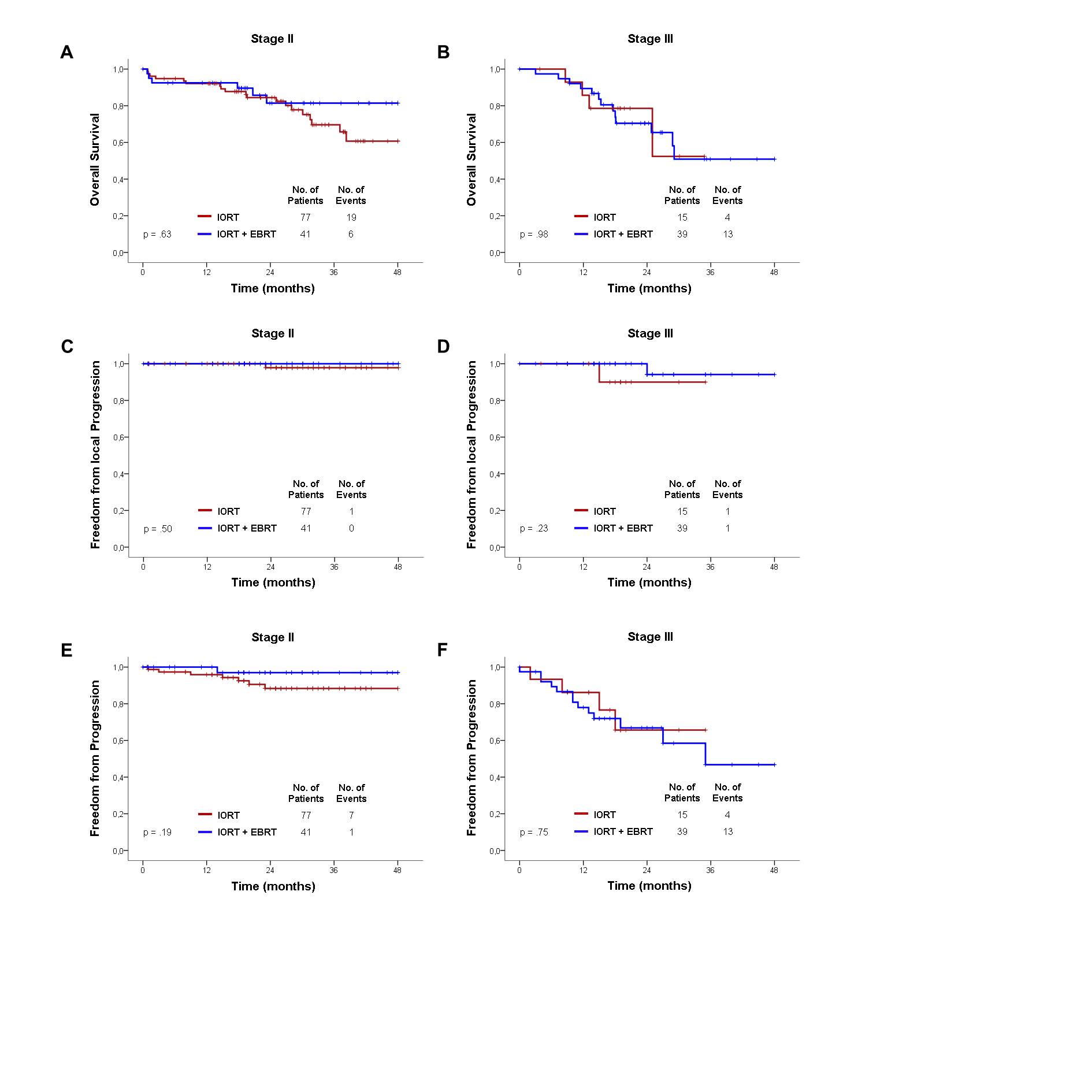

Supplement: Supplementary file 1 — Additional file 1 A1. Stage II or III rectal cancer subgroups. (A, B) Overall survival. (C, D) Freedom from local progression. (E, F) Freedom from local or distant progression. [file 13014_2020_1458_MOESM1_ESM.jpg]
